# Editorial: The Canonical and Non-Canonical Endocannabinoid System as a Target in Cancer and Acute and Chronic Pain

**DOI:** 10.3389/fphar.2020.00312

**Published:** 2020-03-11

**Authors:** Marialessandra Contino, Peter J. McCormick

**Affiliations:** ^1^Dipartimento di Farmacia-Scienze del Farmaco, Università degli Studi di Bari, Bari, Italy; ^2^William Harvey Research Institute, Bart's and The London School of Medicine and Dentistry, Queen Mary University of London, London, United Kingdom

**Keywords:** cancer, cannabinoid receptors (CB1 and CB2), ECS endocannabinoid system, pain, cannabinoid ligands

The endocannabinoid system (ECS) comprises the canonical receptor subtypes CB1R and CB2R and endocannabinoids (anandamide, AEA and 2-arachidonoylglycerol, 2-AG), and a “non-canonical” extended signaling network consisting of: (i) other fatty acid derivatives; (ii) the defined “ionotropic cannabinoid receptors” (TRP channels); other GPCRs (GPR55, PPARα); (iii) enzymes involved in the biosynthesis and degradation of endocannabinoids (FAAH and MAGL); and (iv) protein transporters (FABP family) (Pisanti et al., 2013; Iannotti et al., 2016).The ECS is currently a hot topic due to its involvement in cancer and pain.

High CB1R expression correlates with poor prognosis in different type of cancers including prostate, pancreatic, colorectal, and ovarian cancer (Michalski et al., 2008; Cipriano et al., 2013; Jung et al., 2013; Messalli et al., 2014); while high CB2R expression correlated to poor prognosis in HER2-positive breast cancer (Blasco-Benito et al., 2019). Endocannabinoids such as AEA and 2-AG were found upregulated in different tumors (colorectal carcinomas) compared to healthy subjects (Pyszniak et al., 2016).Despite these changes there have been variable mechanisms suggested for these endocannabinoids in terms of their antitumorigenic activity. The antiproliferative effect induced by AEA in prostate and breast cancers has been reported to be due to CB1R activation (Grimaldi and Capasso, 2011);while the apoptosis induced by R(+)-methanandamide in lymphoma cells is reported to be due to the activation of both CB1R and CB2R (Gustafsson et al., 2008).While its anticancer effect in cervical and lung tumors may be from other pathways (Eichele et al., 2009).The antiproliferative effect of 2-AG appears dependent on pathways involving CB1R-mediated p42/44 MAPK and AKT signaling. Recent studies have demonstrated a link between TRPV2 and CBD-induced autophagy in glioblastoma cells and CB2R-GRP55 heteromers as a cause of cancer cell proliferation have been found (Moreno et al., 2014; Nabissi et al., 2015).

For pain, the ECS plays a role at different points in the nociception axis. AEA and 2-AG elicit long-term depression of both excitatory and inhibitory synapses increasing neural circuit output. Endocannabinoid/TRPV signaling induces the sensitization of the shortening reflex while CB1 and CB2 receptors are targeted in the treatment of pain.

The current Research Topic highlights various ways the ECS can impact cancer and pain.

Ramer et al. review the anticancer potential of the canonical and noncanonical endocannabinoid system. The authors highlight the regulation of the two canonical receptor subtypes CB1R and CB2R in malignant tissue, emphasizing the involvement in cancer onset and progression of the biosynthetic and degradation enzymes.

Morales and Jagerovic provide a much needed summary of cannabinoid ligands as promising antitumor agents in a wide variety of tumors, in contrast to their palliative applications. In their article, the authors classify cannabinoids with anticancer potential in endocannabinoids, phytocannabinoids, and synthetic cannabinoids (arylpyrazoles, aminoalkylindoles, quinones, naphthyridine, and others) reporting the targeted tumor and the corresponding mechanism of action of each study these findings.

Moreno et al. in their review explored the value of cannabinoid receptor heteromers as potential new targets for anti-cancer therapies and as prognostic biomarkers, showing the potential of the endocannabinoid network in the anti-cancer setting as well as the clinical and ethical pitfalls behind it.

As for the antinociceptive potential, Belardo et al. reported a study performed on cannabidiol (CBD), the major non-psychoactive constituent of *Cannabis sativa*, in traumatic brain injury (TBI). In their research article, the authors evaluated the CBD effects on the neurological dysfunctions associated with the TBI demonstrating the ability of oral CBD to prevent allodynia and neurological dysfunctions in a mouse model of mild TBI.

Jones et al. evaluated the therapeutic effect of indomethacin morpholinamide (IMMA), a novel substrate-selective COX-2 inhibitor, to alleviate hyperalgesia and mechanical allodynia in the chronic constriction injury (CCI) mouse model. They observed that IMMA induced anti-nociceptive effects through multiple mechanisms including CB2 receptor activation.

As an ensemble, these studies provide further fuel to the discussion and underline the potential for targeting the ECS at multiple levels to treat certain cancers and for pain relief. Importantly, they also help to move the focal point of the discussion beyond THC, CBD, and the cannonical receptors. Several of these reports either review or provide data to support the use of/targeting of other members of the ECS system as well as alternative natural products beyond THC and CBD. In summary, we hope that this collection of articles continues to drive research in what is proving to be a an important area of research.

## Author Contributions

All authors listed have made substantial, direct, and intellectual contribution to the work and approved it for publication.

## Conflict of Interest

The authors declare that the research was conducted in the absence of any commercial or financial relationships that could be construed as a potential conflict of interest.
